# Combined MR-assisted motion and partial volume effects corrections – impact on PET data quantification

**DOI:** 10.1186/2197-7364-1-S1-A38

**Published:** 2014-07-29

**Authors:** Ciprian Catana, Daniel B Chonde, Kevin T Chen, David Izquierdo-Garcia, Spencer Bowen, Jacob Hooker, Joshua Roffman

**Affiliations:** Athinoula A. Martinos Center for Biomedical Imaging, Department of Radiology, Massachusetts General Hospital and Harvard Medical School, Charlestown, MA USA; Program in Biophysics, Harvard University, Cambridge, MA USA; Department of Health Sciences and Technology, Massachusetts Institute of Technology, Cambridge, MA USA; Psychiatry Department, Massachusetts General Hospital, Kragujevac, MA USA

Our goal in this study was to characterize the combined effect of MR-assisted motion correction (MC) and partial volume effects correction (PVEC) on the estimation of [^11^C]NNC112 binding potential (BP) in healthy volunteers.

29 subjects were scanned on the Siemens 3T MR-BrainPET scanner prototype. Emission data were acquired in list mode format for 90-minutes following the i.v. administration of ~8 mCi of [^11^C]NNC112. The head attenuation map was obtained from the MPRAGE data using an atlas-based method. Head motion estimates were derived from the MR data and used to correct the PET data in LOR space before image reconstruction [1]. PVEC was applied to the motion corrected data using the region-based voxel-wise (RBV) method [2] and regions of interest (ROIs) defined from the MPRAGE images using FreeSurfer and the measured point-spread function [3]. BPnd for each of the ROIs was estimated in PMOD using the simplified reference tissue kinetic (SRTM) model and the cerebellum as a reference tissue.

Maximum translations of up to 9 mm and rotations of up to 12 degrees have been observed in this group of subjects (Figure 1). Less variability in the tissue time activity curves (TACs) was noted after MC (the curves before and after MC for a representative subject are shown in Figure 2). The percentage changes in BPnd after MC and PVEC revealed both under- and overestimation in the ROIs analyzed (Figure 3). The cumulative effect exceeded 100% for some of the structures analyzed.

**Figure 1:**
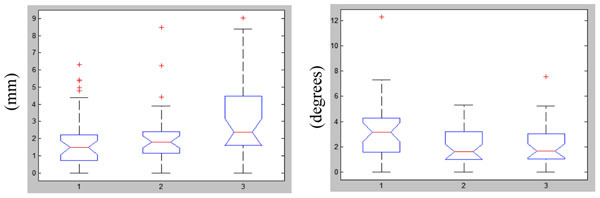
Maximum translations (left) and rotations (right) measured in 29 healthy volunteers scanned for 90 minutes

**Figure 2:**
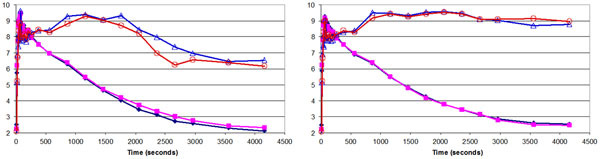
TACs in the left and right caudate and cerebellar cortices before (left) and after (right) MR-assisted motion correction

**Figure 3:**
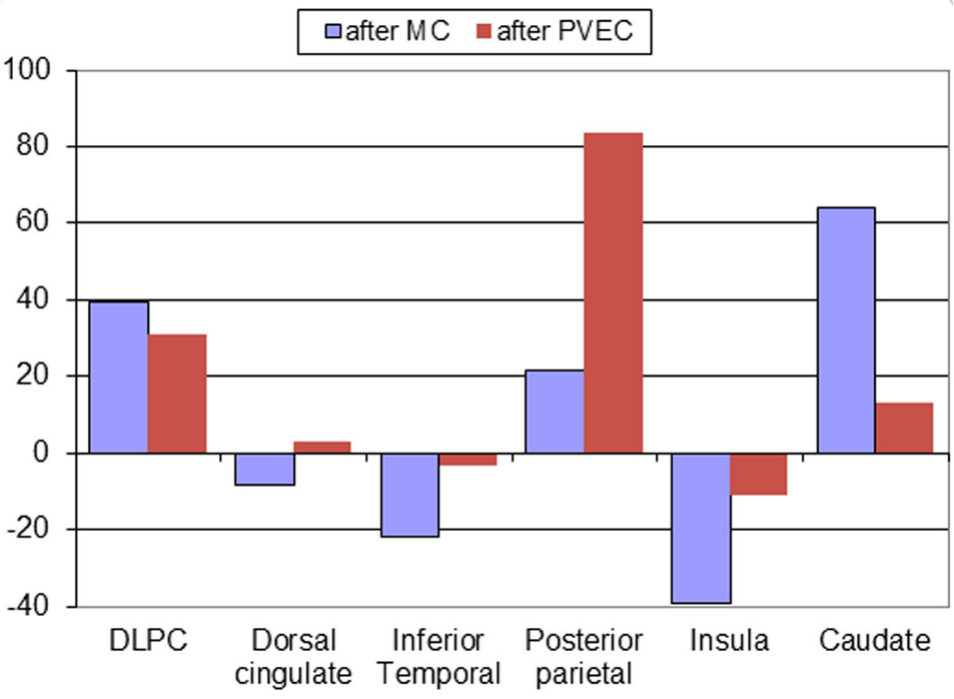
Percent change in NNC112 BPnd after motion and partial volume effects corrections

Significant motion and PVE were observed in all the subjects, biasing the PET estimates. The combined effect is difficult to predict, depending on the size and location of the structure of interest and patient compliance. Without addressing these issues, the value of the BPnd’s derived from these data is questionable.

## References

[1] Catana C, Benner T, van der Kouwe A (2011). MRI-assisted PET motion correction for neurologic studies in an integrated MR-PET scanner. J Nucl Med.

[2] Thomas BA, Erlandsson K, Modat M (2011). The importance of appropriate partial volume correction for PET quantification in Alzheimer's disease. Eur J Nucl Med Mol Imaging.

[3] Bowen SL, Byars LG, Michel CJ, Chonde DB, Catana C (2013). Influence of the partial volume correction method on (18)F-fluorodeoxyglucose brain kinetic modelling from dynamic PET images reconstructed with resolution model based OSEM. Phys Med Biol.

